# Evaluating the Effectiveness of Basic Life Support (BLS) and Advanced Cardiovascular Life Support (ACLS) Training for Family Medicine Residents in Saudi Arabia

**DOI:** 10.7759/cureus.73637

**Published:** 2024-11-13

**Authors:** Renad Althobaiti, Rana Aldhahi, Mohammed Althobaiti, Lena S AlSaleem, Hamad Alkhaldi, Nafea Almutairi, Ibrahim Ahmed

**Affiliations:** 1 Medicine, Imam Mohammad Ibn Saud Islamic University, Riyadh, SAU; 2 Medicine, King Saud Bin Abdulaziz University for Health Sciences, Riyadh, SAU; 3 Pediatrics, King Faisal Specialist Hospital and Research Center (KFSHRC), Riyadh, SAU; 4 Family Medicine, Imam Mohammad Ibn Saud Islamic University, Riyadh, SAU

**Keywords:** acls, automated external defibrillator (aed), bls training, family medicine residency, out-of-hospital cardiac arrest

## Abstract

Background: This study aims to assess the knowledge level of family medicine physicians in Saudi Arabia, regarding basic life support (BLS) and advanced cardiovascular life support (ACLS). Recognizing the importance of timely and effective resuscitation in emergency medical care, the study explores the physicians' proficiency in key resuscitation concepts and identifies areas for improvement.

Methodology: Conducted as an observational cross-sectional study in Saudi Arabia, the research involved family medicine physicians from major hospital centers. A self-administered questionnaire, adapted from previous studies, was utilized to collect demographic data and assess knowledge related to BLS and ACLS.

Results: The demographic analysis revealed a predominantly male participant base (52.3%), with a majority having clinical practice experience of five years or less (74.4%). The survey highlighted positive trends in BLS course attendance, with 60.5% having undergone training within the last year. However, the study identified gaps in ACLS course attendance, as 37.2% reported never having attended such a course. The knowledge assessment unveiled varying proficiency levels among participants, with critical gaps identified in recognizing the full form of automated external defibrillator (AED) (28.5%) and airway maneuvers (20.9%).

Conclusion: This study highlights the need for ongoing education in resuscitation for family medicine physicians, with targeted interventions to address gaps in AED use and airway management. While BLS course attendance shows proactive engagement, increased focus on ACLS training is necessary to further improve resuscitation skills.

## Introduction

Cardiac arrests and accidents are the most common types of emergencies with serious consequences, with an alarmingly high mortality rate reaching up to 90-98% [1]. The majority of cases happen in out-of-hospital places where there is no competent resuscitation by healthcare providers within the demanding time of 3-5 minutes, thus reducing the chance of survival [2]. Therefore, basic life support/advanced cardiovascular life support (BLS/ACLS) is fundamental to saving lives, particularly among healthcare workers who encounter ill patients every day during their practice [3]. Healthcare professionals are expected to have current knowledge of BLS/ACLS guidelines to revive unresponsive and cardiac arrest patients [4].

Studies have shown the importance of BLS/ACLS in mortality reduction. Proper BLS/ACLS intervention in the first few minutes may double or triple a patient's survival rate. Every minute counts for patients who suffer cardiac events, that is to say, every minute that passes by with no or improper intervention can reduce the BLS/ACLS's effectiveness and set the life of the patient in danger, as the chances of successful resuscitation after sudden cardiac arrest decreases by 7-10% with every minute that is delayed [1,5,6]. BLS includes recognition of signs of sudden cardiac arrest, heart attack, stroke, and foreign body airway obstruction and the performance of cardiopulmonary resuscitation (CPR) and defibrillation with an automated external defibrillator (AED) [1]. A study done by Fatani et al. revealed a gap in BLS knowledge among 150 physicians, despite 99.3% having previous BLS training [2]. This is concerning as it highlights a critical need for enhanced and continuous BLS training programs to ensure that family medicine physicians are well-prepared to respond effectively to emergency situations.

Although several studies have been conducted to evaluate the knowledge and practice of BLS/ACLS among public and medical students, very little is known about it among physicians [7,8]. In Saudi Arabia, the literature is limited with regard to the BLS/ACLS knowledge among healthcare professionals. Nevertheless, there is no detailed information regarding BLS knowledge and attitudes among family medicine residents in Saudi Arabia. Therefore, this study aims to evaluate the level of knowledge and attitudes towards BLS/ACLS among family medicine residents in Saudi Arabia and to suggest remedial measures to tackle any deﬁciencies.

## Materials and methods

A cross-sectional observational study was conducted using an online questionnaire (see Appendices) distributed to major hospital centers across Saudi Arabia, including facilities under the Ministry of Health (MOH), King Faisal Specialist Hospital and Research Center (KFSH), National Guard Health Affairs (NGH), Prince Sultan Military Medical City (PSMMC), and Security Forces Hospital (SFH). Approval was obtained from the Institutional Review Board of Imam Mohammad Ibn Saud Islamic University (approval number: 461/2023) before distributing the questionnaire to the main hospital centers in Saudi Arabia through an online, self-administered survey. The questionnaire used was adapted and modified from Fatani et al. [2]. The study duration was six months between May and October 2023, and the target participants were family medicine physicians in Saudi Arabia. Resident names and contact information were acquired directly from residency program administrators. Informed consent was obtained electronically, detailing the study's purpose, confidentiality measures, and voluntary participation nature.

The criteria for inclusion in this study comprised any male or female family medicine physician actively working in Saudi Arabia, while exclusion criteria were defined by the exclusion of family medicine physicians who had voluntarily left the residency program. The questionnaire employed in data collection encompassed demographic details such as age, residency year, gender, nationality, and current living location. Specific inquiries were tailored to assess the knowledge of family medicine physicians regarding BLS and related topics. Data collection was executed through an online survey distributed to family medicine physicians in designated hospital centers in Saudi Arabia. Prior to the commencement of the study, institutional review board approval was secured to ensure ethical standards were upheld. Rigorous measures were implemented to maintain participant confidentiality, including the anonymization of data, with only aggregate results being reported.

The collected data underwent statistical analysis using IBM SPSS Statistics for Windows, Version 24.0 (Released 2016; IBM Corp., Armonk, New York, United States) and appropriate methods to ascertain the level of knowledge among family medicine physicians regarding BLS. Mean and standard deviation were used for the description of ongoing variables, while frequency and percent were used for describing categorical variables. The chi-squared test was used to assess the differences between participants according to their demographic factors considering their level of knowledge. To assess the knowledge, 15 questions were used where for each question, one correct answer was coded as 1, while other responses were coded as 0. Knowledge levels can be assessed on a scale where scores of 0-10 indicate inadequate knowledge reflecting a limited understanding and significant gaps in the subject matter. Given a range between 0 and 15 where a higher score means better knowledge, participants who scored more than 10 were considered to have adequate knowledge. All statements were considered significant if the p-value was lower than 0.05.

## Results

The study included a total of 172 family medicine physicians with a mean age of 27.22 years old (standard deviation: 2.91). The majority of participants were male (52.3%), with 47.7% being female. Regarding residency year, 33.7% were in their first year, 29.7% in the second, 23.3% in the third, and 13.4% in the fourth. The majority of participants were Saudi nationals (93%), and most resided in the middle region (29.1%). The respondents worked across various centers, with the MOH being the most common (35.5%). Daily workload distribution showed that 34.3% dealt with over 15 patients per session. Regarding medical material review, 32.6% reported studying daily, and 74.4% had less than or equal to five years of clinical practice experience since graduation. The majority had their last BLS and ACLS courses within the last year (60.5% and 32.6%, respectively) (Table 1).

**Table 1:** Demographic factors of the participants presented in frequencies (n) and proportion (%) MOH: Ministry of Health; KFSH: King Faisal Specialist Hospital and Research Center; NGH: National Guard Health Affairs; PSMMC: Prince Sultan Military Medical City; SFH: Security Forces Hospital; BLS: basic life support; ACLS: advanced cardiovascular life support

		Frequency and proportion n=172 (%)
Gender	Male	90 (52.3%)
	Female	82 (47.7%)
Residency year	1	58 (33.7%)
	2	51 (29.7%)
	3	40 (23.3%)
	4	23 (13.4%)
Nationality	Saudi	160 (93%)
	Non-Saudi	12 (7%)
Living at	Northern region	29 (16.9%)
	Southern region	5 (2.9%)
	Eastern region	39 (22.7%)
	Western region	49 (28.5%)
	Middle region	50 (29.1%)
Which center are you working in?	MOH	61 (35.5%)
	KFSH	15 (8.7%)
	NGH	8 (4.7%)
	Private sector	22 (12.8%)
	PSMMC	13 (7.6%)
	SFH	12 (7%)
	Others	41 (23.8%)
What is your daily workload (per session)?	0-5 patients	9 (5.2%)
	6-10 patients	52 (30.2%)
	11-15 patients	52 (30.2%)
	>15 patients	59 (34.3%)
How many times do you read/study medical material?	Every month or more	15 (8.7%)
	Every 3 weeks	15 (8.7%)
	Every 2 weeks	27 (15.7%)
	Weekly	59 (34.3%)
	Daily	56 (32.6%)
What is your experience (in years) in clinical practice since medical school graduation?	=5 years	128 (74.4%)
	6-10 years	38 (22.1%)
	11-15 years	4 (2.3%)
	16-20 years	1 (0.6%)
	>20 years	1 (0.6%)
Last BLS course?	Never	1 (0.6%)
	4 years	8 (4.7%)
	2-4 years	59 (34.3%)
	Last year	104 (60.5%)
Last ACLS course?	Never	64 (37.2%)
	4 years	17 (9.9%)
	2-4 years	35 (20.3%)
	Last year	56 (32.6%)

Responses to questions assessing knowledge about BLS revealed that 55.8% correctly identified the American Heart Association as the advocate for BLS. For the full form of AED, 28.5% recognized it as an automated external defibrillator. In scenarios of an unconscious person, 55.2% correctly identified checking for a response as the first step. After finding no pulse, 54.7% correctly stated that the next step is to call for help and get an AED. In child CPR with two persons, 54.1% correctly identified the compression-to-ventilation ratio as 15:2. For adult CPR, 77.3% recognized the correct compression-to-ventilation ratio as 30:2. The majority (62.2%) knew that pulse should be checked every two minutes during CPR. Maneuvers to open the airway were correctly identified by 20.9% for jaw thrust. In situations where there is no pulse between CPR, 72.7% correctly suggested continuing CPR. The order of steps for initially starting CPR was correctly identified by 25.6% as Circulation, Airway, and Breathing (C-A-B). The majority (52.9%) correctly identified that a pulse check during BLS can take 5-10 seconds. For the drug appropriately given in pulseless electrical activity, 19.8% correctly identified Inj. Adrenaline 1 mg 1/10,000. The ideal route of administration of adrenaline during cardiac arrest, identified as IV, was known by 76.2%. Ventricular fibrillation (VF) (39%) was correctly recognized as a shockable electrocardiogram (ECG) rhythm in cardiac arrest. In ACLS, 29.1% and 24.4% correctly identified that both vasopressin and atropine are not used to revive cardiac arrest (Table 2).

**Table 2:** The questions used to assess the level of knowledge presented in frequencies (n) and proportion (%) CPR: cardiopulmonary resuscitation; BLS: basic life support; ECG: electrocardiogram; ACLS: advanced cardiovascular life support; AED: automated external defibrillator; IV: intravenous; IM: intramuscular; SC: subcutaneous; VF: ventricular fibrillation; VT: ventricular tachycardia; PEA: pulseless electrical activity

		Frequency and proportion n=109 (%)
BLS is advocated by?	Do not know	16 (9.3%)
	American Heart Association*	96 (55.8%)
	American Thoracic Society	12 (7%)
	Academic Emergency Medicine	15 (8.7%)
	World Health Organization	33 (19.2%)
Full form of AED is?	Do not know	27 (15.7%)
	Automated external defibrillator*	49 (28.5%)
	Automated electric defibrillator	41 (23.8%)
	Automatic electronic defibrillator	42 (24.4%)
	Autonomous electric defibrillator	13 (7.6%)
What is the first step to do when you see a person lying unconscious in a safe place?	Check for response*	95 (55.2%)
	Check pulse	19 (11%)
	Call for help	51 (29.7%)
	Give rescue breaths	7 (4.1%)
What will you do next after you find the patient has no pulse in the above scenario?	Call for help/get AED*	94 (54.7%)
	Check the response	14 (8.1%)
	Start CPR	61 (35.5%)
	Give rescue breaths	3 (1.7%)
In child CPR with two persons, the ratio of compression to ventilation is?	Do not know	4 (2.3%)
	15:2*	93 (54.1%)
	15:1	35 (20.3%)
	30:2	32 (18.6%)
	3:1	8 (4.7%)
The ratio of compression to ventilation for adult CPR is?	Do not know	5 (2.9%)
	30:2*	133 (77.3%)
	15:2	16 (9.3%)
	15:1	14 (8.1%)
	8:1	4 (2.3%)
When are you supposed to check for pulse in BLS during CPR?	Do not know	7 (4.1%)
	Every 2 minutes*	107 (62.2%)
	Every 3 minutes	9 (5.2%)
	Every 1 minute	29 (16.9%)
	Every 5 minutes	20 (11.6%)
What are the maneuvers to open the airway in an unresponsive patient?	Do not know	6 (3.5%)
	Jaw thrust*	36 (20.9%)
	Head tilt	90 (52.3%)
	Chin lift	36 (20.9%)
	Neck flexion	4 (2.3%)
What is the next step to do for a collapsed patient if there is no pulse between CPR?	Do not know	16 (9.3%)
	Continue CPR*	125 (72.7%)
	Give chest compressions only	12 (7%)
	Check pupil	12 (7%)
	Ventilate only	7 (4.1%)
In BLS guidelines, the order of steps for initially starting CPR is?	Do not know	4 (2.3%)
	Circulation, Airway, and Breathing (C-A-B)*	44 (25.6%)
	Airway, Breathing, and Circulation (A-B-C)	98 (57%)
	Airway, Circulation, and Breathing (A-C-B)	23 (13.4%)
	Breathing, Circulation, and Airway (B-C-A)	3 (1.7%)
In BLS, pulse check can take how much time?	Do not know	15 (8.7%)
	5-10 seconds*	91 (52.9%)
	1-5 seconds	36 (20.9%)
	10-20 seconds	13 (7.6%)
	30-60 seconds	17 (9.9%)
The drug which is appropriately given in pulseless electrical activity is?	Do not know	32 (18.6%)
	Inj. Adrenaline 1 mg 1/10,000*	34 (19.8%)
	Inj. Adrenaline 1 mg 1/1,000	46 (26.7%)
	Inj. Atropine 0.5 mg	18 (10.5%)
	Inj. Atropine 1.0 mg	42 (24.4%)
What is the ideal route of administration of adrenaline during cardiac arrest?	Do not know	13 (7.6%)
	IV*	131 (76.2%)
	IM	18 (10.5%)
	Intracardiac	8 (4.7%)
	SC	2 (1.2%)
The shockable ECG rhythms in cardiac arrest are?	Do not know	39 (22.7%)
	VF*	67 (39%)
	Asystole	16 (9.3%)
	Pulseless VT	40 (23.3%)
	PEA	10 (5.8%)
Which drug is not used in ACLS to revive a cardiac arrest?	Do not know	37 (21.5%)
	Adrenaline	18 (10.5%)
	Vasopressin*	50 (29.1%)
	Amiodarone	25 (14.5%)
	Atropine*	42 (24.4%)

**Figure 1:**
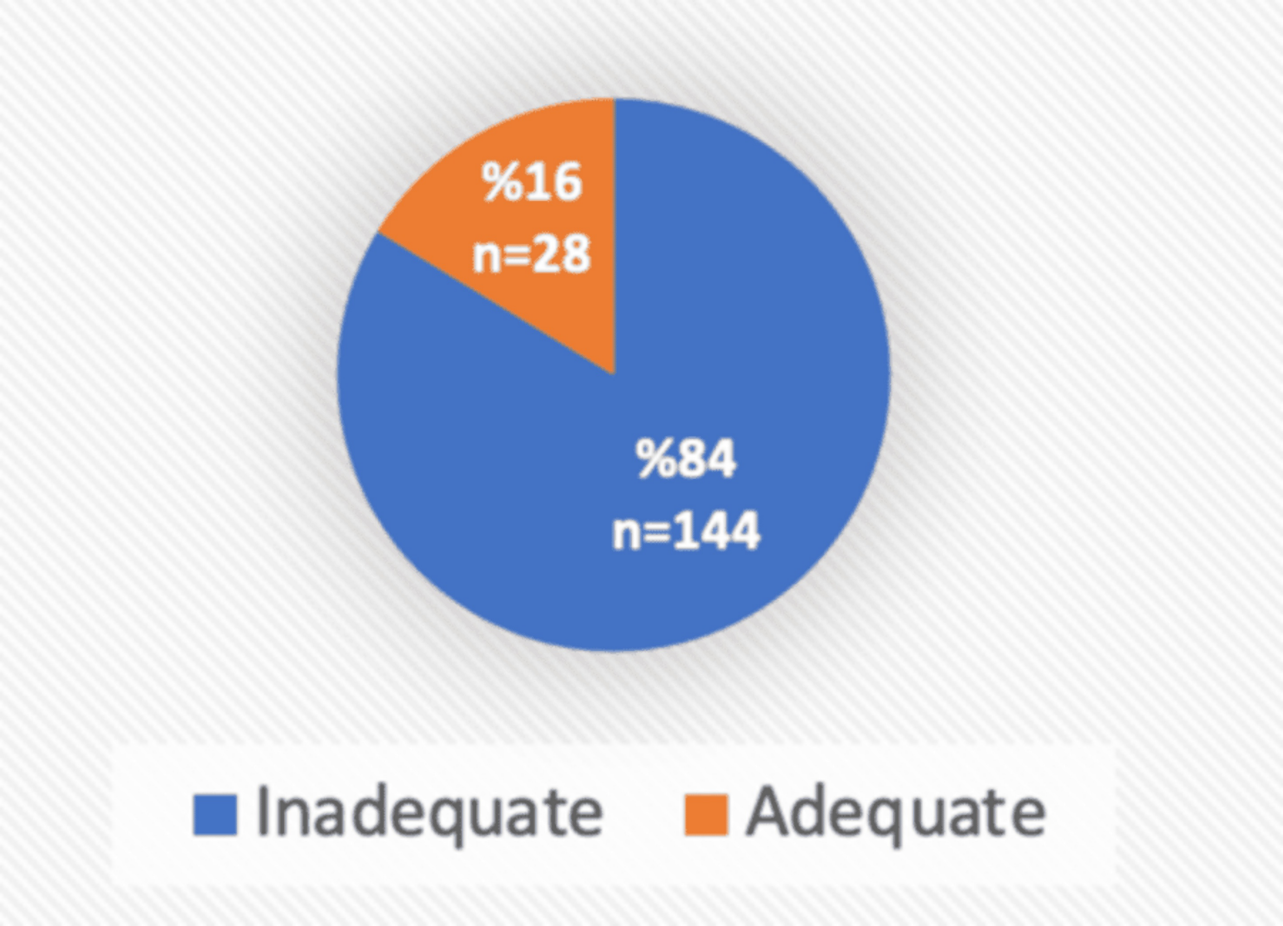
The level of knowledge among the participants

Among the participants, 83.7% had inadequate knowledge, while 16.3% demonstrated adequate knowledge by answering 10 or more questions correctly (Figure 1).

Analyzing the relationship between knowledge and demographic factors revealed certain trends. Notably, those residing in the northern and southern regions had a significantly higher percentage of inadequate knowledge (100%, p=0.000). Additionally, family medicine physicians working in the MOH demonstrated a higher percentage of inadequate knowledge 50 (82%, p=0.038). Other demographic factors did not show significant associations with knowledge levels (Table 3).

**Table 3:** The relation between knowledge and demographic factors. Data are represented as frequencies (N), proportions (%), or mean±SD. Statistical significance was determined using a p-value threshold, with p<0.05 considered significant and p<0.001 considered highly significant MOH: Ministry of Health; KFSH: King Faisal Specialist Hospital and Research Center; NGH: National Guard Health Affairs; PSMMC: Prince Sultan Military Medical City; SFH: Security Forces Hospital; BLS: basic life support; ACLS: advanced cardiovascular life support

		Knowledge			
		Inadequate	Adequate	Chi-squared test	P-value
		Frequency and proportion n=144 (%)	Frequency and proportion n=28 (%)		
Gender	Male	71 (78.9%)	19 (21.1%)	χ²=3.24	0.072
	Female	73 (89%)	9 (11%)		
Residency year	1	50 (86.2%)	8 (13.8%)	χ²=1.5; χ²=1.54	0.662
	2	43 (84.3%)	8 (15.7%)		
	3	31 (77.5%)	9 (22.5%)		
	4	20 (87%)	3 (13%)		
Nationality	Saudi	132 (82.5%)	28 (17.5%)	χ²=2.51	0.113
	Resident in Saudi Arabia	12 (100%)	0 (0%)		
Living at	Northern region	29 (100%)	0 (0%)	χ²=26.99	0.000*
	Southern region	5 (100%)	0 (0%)		
	Eastern region	28 (71.8%)	11 (28.2%)		
	Western region	47 (95.9%)	2 (4.1%)		
	Middle region	35 (70%)	15 (30%)		
Which center are you working in?	MOH	50 (82%)	11 (18%)	χ²=12.23	0.038*
	KFSH	10 (66.7%)	5 (33.3%)		
	NGH	7 (87.5%)	1 (12.5%)		
	Private sector	21 (95.5%)	1 (4.5%)		
	PSMMC	12 (92.3%)	1 (7.7%)		
	SFH	7 (58.3%)	5 (41.7%)		
	Others	37 (90.2%)	4 (9.8%)		
What is your daily workload (per session)?	0-5 patients	8 (88.9%)	1 (11.1%)	χ²=3.03	0.387
	6-10 patients	42 (80.8%)	10 (19.2%)		
	11-15 patients	41 (78.8%)	11 (21.2%)		
	>15 patients	53 (89.8%)	6 (10.2%)		
How many times do you read/study medical material?	Every month or more	13 (86.7%)	2 (13.3%)	χ²=7.03	0.108
	Every 3 weeks	11 (73.3%)	4 (26.7%)		
	Every 2 weeks	24 (88.9%)	3 (11.1%)		
	Weekly	54 (91.5%)	5 (8.5%)		
	Daily	42 (75%)	14 (25%)		
What is your experience (in years) in clinical practice since medical school graduation?	=5 years	105 (82%)	23 (18%)	χ²=0.46	0.790
	6-10 years	33 (86.8%)	5 (13.2%)		
	11-15 years	4 (100%)	0 (0%)		
	16-20 years	1 (100%)	0 (0%)		
	>20 years	1 (100%)	0 (0%)		
Last BLS course?	Never	1 (100%)	0 (0%)	χ²=1.02	0.618
	4 years	7 (87.5%)	1 (12.5%)		
	2-4 years	52 (88.1%)	7 (11.9%)		
	Last year	84 (80.8%)	20 (19.2%)		
Last ACLS course?	Never	52 (81.3%)	12 (18.8%)	χ²=0.28	0.868
	4 years	15 (88.2%)	2 (11.8%)		
	2-4 years	29 (82.9%)	6 (17.1%)		
	Last year	48 (85.7%)	8 (14.3%)		

## Discussion

The results of this study provide valuable insights into the knowledge levels of family medicine physicians in Riyadh, Saudi Arabia, regarding BLS. Understanding the strengths and weaknesses in their knowledge is crucial for improving training programs and ensuring the delivery of high-quality healthcare. 

The study provides insightful information about the frequency of BLS and ACLS courses among family medicine physicians in Riyadh. Notably, the majority of participants had attended a BLS course within the last year (60.5%), reflecting a positive trend in maintaining up-to-date resuscitation skills and which is higher than reported in studies reported in Oman (35.2%) [6], Egypt (27%) [9], Saudi Arabia (22.5%) [10], India (18.9%) [11], and Pakistan (14.7%) [12]. This finding suggests a proactive approach among family medicine physicians in Riyadh, as regular BLS training is crucial for ensuring healthcare professionals are well-prepared to respond to emergencies [13]. In contrast, the rate of ACLS course attendance showed variations, with 37.2% of participants reporting never having taken an ACLS course. This percentage is higher than expected, given the critical role of ACLS in managing more complex cardiac emergencies. The low rate of ACLS training may raise concerns about the preparedness of family medicine physicians to handle advanced resuscitation scenarios. The reasons behind this lower participation rate in ACLS courses merit further investigation.

In examining the least knowledgeable items among family medicine physicians, certain critical aspects of BLS emerged where gaps in understanding were evident. Notably, the low recognition (28.5%) of the full form of AED is a cause for concern. AEDs play a pivotal role in early defibrillation during sudden cardiac arrest, significantly influencing patient outcomes [14]. The lack of familiarity with the term raises questions about the physicians' preparedness to utilize this life-saving device effectively [15]. Proper utilization of AEDs requires swift and accurate responses, and the deficiency in recognizing their full form may hinder the timely initiation of crucial interventions, potentially compromising patient survival [16].

Similarly, the item pertaining to maneuvers to open the airway in an unresponsive patient saw a low correct response rate (20.9%). The correct technique, jaw thrust, is fundamental in ensuring a patent airway during resuscitation efforts [17,18]. Inaccuracies in airway management can have severe consequences, particularly in cases of respiratory distress or obstruction [19]. Failure to employ the correct maneuver may impede adequate oxygenation, exacerbating the patient's condition and leading to adverse outcomes [19].

The lack of knowledge in these critical areas has direct implications for patient health and outcomes. In scenarios requiring immediate intervention, such as cardiac arrest, delays or errors in utilizing essential equipment like AEDs can contribute to a decline in the effectiveness of resuscitative efforts [20]. Early defibrillation is a cornerstone of successful resuscitation, and unfamiliarity with AEDs may result in avoidable delays, potentially reducing the chances of restoring normal cardiac rhythm and survival.

Similarly, deficiencies in airway management techniques can lead to inadequate oxygenation, exacerbating hypoxia and compromising overall patient well-being [19]. Proper airway maneuvers are essential not only in cardiac arrest scenarios but also in a broader spectrum of emergencies involving respiratory distress [21]. The repercussions of incorrect airway management may manifest as prolonged hypoxia, increased risk of complications, and, in extreme cases, irreversible neurological damage [19].

Conversely, certain aspects of BLS demonstrated a high level of knowledge among family medicine physicians. Notably, the majority correctly identified the compression-to-ventilation ratio for adult CPR as 30:2 (77.3%) and the compression-to-ventilation ratio for pediatric CPR as 15:2 (54.1%). This knowledge is pivotal in providing effective chest compressions and ventilation during CPR. Understanding and implementing the correct compression-to-ventilation ratio are fundamental in maintaining adequate perfusion and oxygenation, contributing significantly to successful resuscitation outcomes [22,23].

Additionally, a substantial proportion (62.2%) correctly identified the recommended frequency for checking the pulse during BLS as every two minutes. This adherence to established guidelines is crucial in ensuring the timely assessment of the patient's response to interventions. Consistent pulse checks enable healthcare providers to adjust their approach promptly, tailoring resuscitative efforts based on the patient's evolving condition [22,24].

High levels of knowledge in these areas directly translate into improved patient outcomes. Adherence to recommended compression-to-ventilation ratios ensures the delivery of high-quality CPR, optimizing blood circulation and oxygenation [25]. This, in turn, increases the likelihood of restoring spontaneous circulation and preserving vital organ function. The correct understanding of the pulse-check frequency is equally critical in preventing delays in recognizing changes in the patient's status. Prompt assessments enable healthcare providers to identify the effectiveness of interventions, make informed decisions, and adjust their approach accordingly. 

Examining the knowledge levels based on demographic factors, a notable trend emerged in the regional distribution. Family medicine physicians residing in the northern and southern regions exhibited a 100% rate of inadequate knowledge, a finding that demands attention and further investigation. This regional disparity may be attributed to variations in training programs, access to educational resources, or specific challenges faced by healthcare professionals in that area. Addressing these regional differences is crucial for ensuring standardized and comprehensive training across all regions. The results showed that gender had no apparent effect on knowledge level. In Saudi Arabia, a similar finding was reported [26], although another study in France found a difference between males and females [27]. Similarly, family medicine physicians working in the MOH demonstrated a higher percentage of inadequate knowledge (82%). This finding could be associated with variations in training curricula, resource availability, or the emphasis placed on continuous medical education within different healthcare settings. The MOH, being a major healthcare provider, should consider targeted interventions to address knowledge gaps among its staff, potentially through tailored educational programs and regular updates.

Limitation

It is essential to acknowledge certain limitations in our study. Firstly, the sample size was relatively small, which might limit the generalizability of our findings to the broader population of family medicine physicians in the region. Additionally, the cross-sectional nature of the study design implies a snapshot of knowledge at a specific point in time, making it challenging to establish causation or temporal relationships. Moreover, the representativeness of the sample may be affected by potential selection bias, as physicians who volunteered to participate might differ systematically from those who chose not to.

## Conclusions

Based on the findings of this study, it is evident that there are considerable knowledge deficits in the BLS practices among Riyadh family medicine residents such as AED use and airway management. It emphasizes the importance of continuing education and the need for joint training programs for improvement in patients' outcomes in emergency situations. These findings lay the groundwork for future educational projects exploring the perceptive and educational potentials of family medicine residents practicing in and around Riyadh.

## Appendices

**Table 4:** BLS and ACLS research questions and answers CPR: cardiopulmonary resuscitation; BLS: basic life support; ECG: electrocardiogram; ACLS: advanced cardiovascular life support; AED: automated external defibrillator; IV: intravenous; IM: intramuscular; SC: subcutaneous; VF: ventricular fibrillation; VT: ventricular tachycardia; PEA: pulseless electrical activity

Question	Options	Answer
BLS is advocated by?	a. American Thoracic Society	d. American Heart Association
	b. World Health Organization	
	c. Academic Emergency Medicine	
	d. American Heart Association	
	e. Don't know	
Full form of AED is?	a. Automatic electronic defibrillator	c. Automated external defibrillator
	b. Autonomous electric defibrillator	
	c. Automated external defibrillator	
	d. Automated electric defibrillator	
	e. Don't know	
What is the first step to do when you see a person lying unconscious in a safe place?	a. Call for help	b. Check for response
	b. Check for response	
	c. Give rescue breaths	
	d. Check pulse	
	e. Don't know	
What will you do next after you find the patient has no pulse in the above scenario?	a. Call for help/get AED	a. Call for help/get AED
	b. Check for response	
	c. Give rescue breaths	
	d. Start CPR	
	e. Don't know	
In child CPR with two persons, the ratio of compression to ventilation is?	a. 15:1	d. 15:2
	b. 3:1	
	c. 30:2	
	d. 15:2	
	e. Don't know	
The ratio of compression to ventilation for adult CPR is?	a. 30:2	a. 30:2
	b. 8:1	
	c. 15:1	
	d. 15:2	
	e. Don't know	
When are you supposed to check for pulse in BLS during CPR?	a. Every 1 minute	b. Every 2 minutes
	b. Every 2 minutes	
	c. Every 3 minutes	
	d. Every 5 minutes	
	e. Don't know	
What are the maneuvers to open the airway in an unresponsive patient?	a. Head tilt	a. Head tilt
	b. Chin lift	
		b. Chin lift
	c. Neck flexion	
	d. Jaw thrust	d. Jaw thrust
	e. Don't know	
What is the next step to do for a collapsed patient if there is no pulse between CPR?	a. Ventilate only	b. Continue CPR
	b. Continue CPR	
	c. Give chest compressions only	
	d. Check pupil	
	e. Don't know	
In BLS guidelines, the order of steps for initially starting CPR is?	a. Circulation, Airway, and Breathing (C-A-B)	a. Circulation, Airway, and Breathing (C-A-B)
	b. Airway, Circulation, and Breathing (A-C-B)	
	c. Airway, Breathing, and Circulation (A-B-C)	
	d. Breathing, Circulation, and Airway (B-C-A)	
	e. Don't know	
In BLS, pulse check can take how much time?	a. 1-5 seconds	b. 5-10 seconds
	b. 5-10 seconds	
	c. 10-20 seconds	
	d. 30-60 seconds	
	e. Don't know	
The drug which is appropriately given in pulseless electrical activity is?	a. Inj. Atropine 0.5 mg	c. Inj. Adrenaline 1 mg 1/10,000
	b. Inj. Atropine 1 mg	
	c. Inj. Adrenaline 1 mg 1/10,000	
	d. Inj. Adrenaline 1 mg 1/1,000	
	e. Don't know	
What is the ideal route of administration of adrenaline during cardiac arrest?	a. IV	a. IV
	b. IM	
	c. Intracardiac	
	d. SC	
	e. Don't know	
The shockable ECG rhythms in cardiac arrest are?	a. PEA	b. VF
	b. VF	c. Pulseless VT
	c. Pulseless VT	
	d. Asystole	
	e. Don't know	
Which drug is not used in ACLS to revive a cardiac arrest?	a. Atropine	a. Atropine
	b. Adrenaline	
	c. Amiodarone	
	d. Vasopressin	
	e. Don't know	
